# The Translocator Protein (TSPO) in Mitochondrial Bioenergetics and Immune Processes

**DOI:** 10.3390/cells9020512

**Published:** 2020-02-24

**Authors:** Calina Betlazar, Ryan J. Middleton, Richard Banati, Guo-Jun Liu

**Affiliations:** 1 Human Health, Australian Nuclear Science and Technology Organisation, New Illawarra Road, Lucas Heights, NSW 2234, Australia; rym@ansto.gov.au (R.J.M.); rib@ansto.gov.au (R.B.); 2 Discipline of Medical Imaging & Radiation Sciences, Faculty of Medicine and Health, Brain and Mind Centre, University of Sydney, 94 Mallett Street, Camperdown, NSW 2050, Australia

**Keywords:** neuroinflammation, translocator protein, microglia, reactive oxygen species, mitochondria

## Abstract

The translocator protein (TSPO) is an outer mitochondrial membrane protein that is widely used as a biomarker of neuroinflammation, being markedly upregulated in activated microglia in a range of brain pathologies. Despite its extensive use as a target in molecular imaging studies, the exact cellular functions of this protein remain in question. The long-held view that TSPO plays a fundamental role in the translocation of cholesterol through the mitochondrial membranes, and thus, steroidogenesis, has been disputed by several groups with the advent of TSPO knockout mouse models. Instead, much evidence is emerging that TSPO plays a fundamental role in cellular bioenergetics and associated mitochondrial functions, also part of a greater role in the innate immune processes of microglia. In this review, we examine the more direct experimental literature surrounding the immunomodulatory effects of TSPO. We also review studies which highlight a more central role for TSPO in mitochondrial processes, from energy metabolism, to the propagation of inflammatory responses through reactive oxygen species (ROS) modulation. In this way, we highlight a paradigm shift in approaches to TSPO functioning.

## 1. Introduction

The innate immune response is an emergent phenomenon arising from complex molecular and cellular signalling pathways. In the central nervous system (CNS), the innate immune response is orchestrated by microglial cells, capable of adopting differential functional states in response to stressors. The switch from homeostatic microglia to activated, potentially pro-inflammatory, microglia has become an important indicator of pathology in the CNS [1,2]. This switch is also known as M1/M2 polarization, where classically activated pro-inflammatory M1 microglia and alternatively activated M2 microglia lie on opposite ends of a continuum of functional states [3,4]. The pro-inflammatory M1 polarisation of resident microglia is often referred to as “neuroinflammation” to distinguish it from inflammatory tissue responses with recruitment of peripheral immunocytes. Neuroinflammation has been described across a wide spectrum of diseases, including psychiatric disorders, neurodegeneration and autoimmune diseases [5,6,7,8,9].

The strong metabolic demand of activated, proliferating microglia is reflected in the functional changes of mitochondria [10,11], and an intrinsic link exists between mitochondrial energetics and the mounting of an inflammatory response [12,13]. The pro-inflammatory M1 phenotype of microglia is underpinned by a switch from oxidative phosphorylation to glycolysis and involves shifts in mitochondrial fission and fusion [13,14]. Mitochondrial proteins are also involved in the signalling transduction pathways that activate inflammatory pathways to release pro-inflammatory factors, and also mediate apoptosis. Facilitating these immune responses is mitochondrial reactive oxygen species (ROS) release and oxidative stress signalling [15,16,17,18,19], as well as the NLRP3 inflammasome complex activation [20]. It is, therefore, important to understand the subcellular drivers of microglial reactivity, which will ultimately aid in the detection and targeting of key mediators of these immune processes.

One of the most widely studied biomarkers of microglial activation is the translocator protein 18 kDa (TSPO). TSPO is a transmembrane protein located primarily on the outer mitochondrial membrane [21,22], and was originally discovered as a peripheral binding site to diazepam [23], hence being long referred to as the peripheral benzodiazepine receptor (PBR). The marked upregulation of TSPO in activated microglia in a broad spectrum of neurobiological diseases has made TSPO a prominent target for positron emission tomography (PET) imaging studies [24]. Concomitantly, a large field of enquiry into TSPO binding agents for diagnostics and therapeutics has also emerged. Indeed, even the earliest description of TSPO upregulation in activated microglia was observed through the increased binding of prototypical TSPO ligands 1-(2-chlorophenyl)-N-methyl-N-(1-methylpropyl)-3-isoquinolinecarboxamide (PK11195) and 4’-Chlorodiazepam (Ro5-4864) in injury models [25,26]. Whilst these ligands are still widely used in studies probing TSPO functioning, newer classes of TSPO ligands have also emerged which will be mentioned throughout this review. Due to the prolific research into TSPO binding agents, several reviews have covered the many TSPO molecular imaging agents and their use in imaging studies in a spectrum of disorders [27,28,29], including neurodegenerative disorders [8,30,31,32,33], psychiatric disorders [34,35,36] and tumours [37].

Despite being widely utilised in imaging studies, the exact functions of TSPO are not well understood. It was long posited that TSPO was primarily involved in the translocation of cholesterol, and thus, steroidogenesis, supported by the strong abundance of TSPO expression in steroidogenic organs [38,39]. This led to the change in nomenclature from “peripheral benzodiazepine receptor” to “translocator protein” [40]. However, sparked by the creation of global TSPO knockout mouse models from independent groups [41,42,43,44], in which mice displayed normal pregnenolone levels, an overall shift in thinking about the functioning of TSPO emerged (several reviews exist which detail the history of TSPO research more comprehensively [45,46,47]). The field of TSPO has since expanded into several different pathways, with many of these lines of enquiry converging on the primary role of TSPO in immunomodulation, cellular bioenergetics, and associated mitochondrial processes. These lines of evidence have been achieved mainly through genetic deletion studies and studies using both older and novel TSPO binding agents. Along with this, several studies are now highlighting the potential neuroprotective properties of TSPO ligands in a broad range of disease or injury models.

In this review, we describe the more recent and growing body of literature surrounding the evolving knowledge of the functions of TSPO. Whilst TSPO has been studied in a broad spectrum of disease states in PET imaging studies, in this review we integrate more direct studies examining the role of TSPO in inflammatory responses, and its more central role in the modulation of mitochondrial energetic processes.

## 2. TSPO in Mitochondrial Processes

### 2.1. TSPO in Mitochondrial Bioenergetics

After a hypothesis first proposed by Anholt (1986), the earliest studies linking the function of TSPO to mitochondrial energy respiration used compounds targeting TSPO on isolated mitochondria from organs strongly expressing TSPO, such as the kidney, adrenals and liver [48,49]. This was followed by studies in a neuroblastoma cell line, where the high affinity ligands PK11195, and to a lesser extent, Ro5-4864, in co-culture resulted in dose-dependent increases in oxygen consumption compared to non-treated controls [50]. It was later found in a glioma cell line that these TSPO ligands can also increase mitochondrial numbers and mitochondrial division, further linking TSPO to mitochondrial dynamics [51]. However, from the earliest studies, the known strong presence of TSPO expression in steroidogenic organs and the ability of cholesterol to bind with high affinity to TSPO led to several years of study into the specific role of TSPO in cholesterol transport and steroidogenesis [39,52,53,54]. As this function continues to produce conflicting evidence, a role for TSPO in steroid synthesis cannot be ruled out, though further data in a range of experimental models is required.

In 2014, independent research groups created global and conditional TSPO knockout models which were found to be viable with no observable abnormalities, and importantly, no effect on steroidogenesis [41,43,55]. It was found, however, that microglia from TSPO knockout animals had lower ATP synthesis and oxygen consumption rates than their wildtype counterparts, suggesting that TSPO deficiency leads to alterations in microglial metabolic activity [41]. Extending on these findings, this group also demonstrated that TSPO overexpression in a human T-cell line increases mitochondrial ATP synthesis, compared to wildtype and empty-plasmid control cells, measured with the luciferin-luciferase bioluminescence assay in permeabilised Jurkat cells with intact mitochondria and cell structure. TSPO overexpressed cells also exhibited increased proliferation rates compared to wildtype and empty-plasmid control cells [56], thus establishing that a key role for TSPO may lie within cellular bioenergetics. 

In more recent years, this path of inquiry has been pursued by multiple groups, using both rodent and human microglia. In the human microglia C20 cell line engineered for TSPO knockout, pregnenolone levels were unchanged in wildtype and knockout cells, including when exposed to TSPO ligands [57]. In agreement with Banati et al. (2014), basal and maximal respiration, as well as ATP-related oxygen consumption, was lower in TSPO knockout cells compared to wildtype cells. This was accompanied by reduced mitochondrial membrane potential and calcium retention [57]. In another recent study, using the rodent microglial cell line BV2, TSPO knockdown cells had altered mitochondrial membrane potential compared to scrambled controls. Furthermore, scramble BV2 cells treated with Ro5-4864 and PK11195 had increased basal respiration and ATP-related respiration [58], demonstrating the ability of TSPO-specific ligands to alter mitochondrial processes of microglia. A very recent and comprehensive study of the role of TSPO in several key mitochondrial functions has demonstrated similar results. TSPO knockout glioma cells were shown to have more mitochondrial fragmentation, increased levels of mitochondrial fission proteins such as FIS1, and decreased complex I activity compared to wildtype cells. TSPO knockout cells also had lower mitochondrial membrane potential, decreased global ATP production, reduced basal and maximal mitochondrial respiratory capacity and higher ROS levels compared to wildtype cells. These findings were also replicated in TSPO knockdown patient-derived stem-like GBM1B cells, demonstrating analogous effects in human cells [59]. Mechanistically, TSPO has been linked to the F_1_F_o_-ATP synthase, the mitochondrial enzyme and protein complex responsible for synthesising cellular ATP. PK11195, Ro5-4864 and protoporphyrin IX (PPIX) have been found to modulate the phosphorylation of subunit c of the F_1_F_o_-ATP synthase in isolated rat brain mitochondria [60]. PK11195 has also been found to inhibit mitophagy in Bcl-2 knockdown HeLa cells by inhibiting activity of the F_1_F_o_-ATP synthase, in a similar way to oligomycin [61]. 

### 2.2. TSPO and Redox Mechanisms

An important by-product of the mitochondrial electron transport chain is the production of free radicals. Significant evidence has emerged linking TSPO to the production and modulation of ROS, important to the functioning of pro-inflammatory microglia [19]. In BV2 microglial cells, PK11195, Ro5-4864 and PPIX exposure in culture increased initial ROS production compared to non-exposed controls, and pre-treatment with an antioxidant blocked this effect [62]. In response to a stressor, TSPO transfected Jurkat cells were more resistant to UV-induced apoptosis, due to delayed membrane depolarisation, reduced caspase-3 activity and reduced superoxide generation compared to wildtype cells [63]. More recently, in a model of Alzheimer’s disease using amyloid precursor protein (APP) overexpressed neuroblastoma cells, newly synthesised imidazoquinazolinone TSPO ligands were able to reduce oxidative injury by decreasing ROS generation in response to H_2_O_2_ stimulation. These ligands could also stabilise mitochondrial respiration under stress conditions, and ultimately reduce levels of amyloid beta formation [64]. These novel ligands have also been found to increase levels of ATP production and stabilise mitochondrial membrane potential unrelated to any effects on steroidogenesis [65]. TSPO ligands have also demonstrated analogous effects in other immune cell types, as PK11195 was shown to reduce basal ROS generation in mouse primary peritoneal macrophages [66], and Ro5-4864 has been shown to preserve mitochondrial membrane potential and reduce ROS production after glucose deprivation stress in astrocytes, compared to controls [67]. 

Interestingly, TSPO and its involvement in ROS modulation has also been linked to mitophagy [17,68], a process which has been implicated in the innate immune response [69]. Through an interaction with voltage-dependent anion channel 1 (VDAC1), TSPO was able to inhibit mitophagy by increasing ROS production in mouse embryonic fibroblasts and canine mammary gland epithelial cells. TSPO was able to modulate levels of LC3B-II activity and the ubiquitination of key proteins involved in mitophagy. TSPO knockdown cells also displayed greater ATP production in response to challenge, and greater glutathione levels, compared to overexpressing cells and controls [68]. The production of ROS is important in the functioning of microglial responses, and in a recent hypothesis proposed by Guilarte et al. (2016), an interaction between TSPO and NOX2, producing ROS, may activate Nrf2 which is involved in redox homeostasis and antioxidant responses. This complex may work to regulate release of ROS in microglia, particularly in states of chronic neuroinflammation where there is prolonged activation causing oxidative damage to surrounding tissue [70]. 

TSPO functioning has also been linked to mitochondrial cell death processes and apoptosis through oxidative stress [71]. PK11195 pre-treatment in U118MG glioblastoma cells was able to prevent cell death induced by cobalt(II) chloride by inhibiting apoptosis, mitochondrial membrane potential collapse and cardiolipin oxidation. These effects were also observed after TSPO knockdown in U118MG cells [72]. TSPO has also been linked to nitric oxide (NO) in the induction of cell death. In U118MG cells, application of PK11195 with the NO donor sodium nitroprusside (SNP) attenuated cell death, counteracted decreases in metabolic activity, and reduced mitochondrial membrane potential collapse compared to SNP or vehicle controls. This effect was similar in TSPO knockdown cells, adding further evidence to the involvement of TSPO in cell death processes [73]. A similar phenomenon has also been demonstrated in a Drosophila model. TSPO knockout Drosophila had reduced gamma radiation-induced apoptosis, as well as increased lifespan compared to wildtype flies. TSPO knockout flies also had decreased oxidative phosphorylation enzymatic activity, increased oxidative stress, and reduced mitochondrial respiration after 1 week [74]. This was also confirmed later in a Drosophila model of alcohol dependence, where flies with conditional TSPO knockout in neurons had increased ROS production in the brain, although this was sex-dependent [75]. 

### 2.3. In Vivo Evidence for TSPO in Mitochondrial Processes

**Figure 1:**
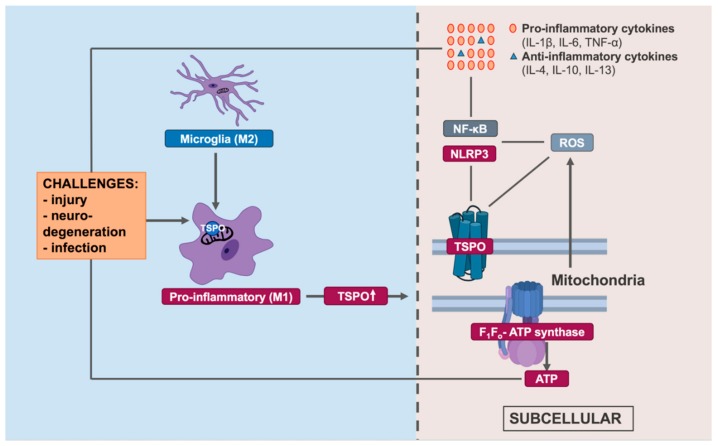
Overview of the translocator protein (TSPO) in the inflammatory responses of microglia and its interaction with mitochondrial processes. Under stress conditions, TSPO is upregulated in activated, pro-inflammatory (M1) microglia. Located on the outer mitochondrial membrane, TSPO interacts with reactive oxygen species (ROS), a key part of the microglial inflammatory response. TSPO also interacts with inflammatory transcriptional pathways including MAPK and the NLRP3 inflammasome, resulting in the release of cytokines. These processes can be modulated by TSPO ligands, and by genetic deletion of TSPO, indicating a key role for TSPO in these processes.

In concordance with this data, other studies using in vivo models have also demonstrated a key role for TSPO in mitochondrial processes, and also in neuroprotection. In a model of postischemia reperfusion in rats, Ro5-4864 pre-treatment reduced ROS levels, reduced the activity of NADPH oxidase, and increased activity of complex 1 and III of the electron transport chain in heart tissue, thereby improving functional recovery [76]. This ligand has also been shown to have mitochondrially-targeted protective properties in a rat model of myocardial ischemia-reperfusion, where Ro5-4864 pre-treatment in hypercholesterolemic rats inhibited cholesterol accumulation and restored oxidative phosphorylation compared to vehicle controls [77]. In an in vivo model of cortical trauma injury in rats, administration of Ro5-4864 after cortical contusion reduced the severity of brain mitochondrial damage as seen under transmission electron microscopy (TEM). This was accompanied by a reduced lactate/pyruvate ratio, indicating less metabolic damage in these animals compared to vehicle treated animals, ultimately leading to improved recovery [78]. Neuroprotective effects of newer TSPO ligands have also been reported. In a rat model of cortical infarction, 2-(2-chlorophenyl) quinazolin-4-yl dimethylcarbamate (2-Cl-MGV) administration after cerebral artery occlusion was found to prevent the collapse of mitochondrial membrane potential and reduce cytochrome c levels in the thalamus of injured rats. 2-Cl-MGV also rescued cognitive impairments and neuronal loss after injury compared to vehicle controls [79]. In a mouse model of spinal cord injury, administration of the TSPO ligand ZBD-2 following injury downregulated TSPO expression, reduced levels of inducible nitric oxide synthase (iNOS) and malondialdehyde (MDA), and increased levels of superoxide dismutase (SOD) in serum, whilst also reducing neuronal loss after injury [80], hence having antioxidative properties. It is therefore apparent that there is much emerging literature surrounding the role of TSPO in mitochondrial processes, including energy metabolism and ROS generation (Table 1). This is also important in the context of immune responses, as these mechanisms are intrinsically involved in the pro-inflammatory responses of microglia (Figure 1). 

## 3. TSPO in the Innate Immune Response

### 3.1. Microglial TSPO Expression and Modulation by TSPO Ligands 

As a prominent molecular imaging biomarker of activated microglia in a range of pathologies, it is generally presumed that TSPO is an indicator of pro-inflammatory, aberrant microglial activation —hence “neuroinflammation”. However, the precise role of TSPO in the inflammatory processes of microglia during active disease states remains unclear. Furthermore, this is complicated by the known complexity of the functional states of microglia, most likely a spectrum from M1/M2 responses, depending on the stimulus [81]. The question of where on the spectrum TSPO is most prominently upregulated has been recently addressed by several studies. In a human microglial cell line, it was recently demonstrated that TSPO is directly involved in the modulation of pro- and anti-inflammatory phenotypes. TSPO mRNA expression was found to be greatest in cells after exposure to pro-inflammatory stimulation with IL-1β and IFN-γ. Differential secretion of pro- and anti-inflammatory cytokines could also be modulated by TSPO ligands and genetic knockdown. This was underpinned by mitochondrial ROS modulation, and NF-κB pathway expression, thereby underscoring TSPO in the mitochondrial processes of inflammatory responses [82]. Similarly, lipopolysaccharide (LPS) stimulation of mouse primary microglia resulted in increased TSPO expression in M1 microglia, though not in IL-4 treated M2 microglia. Thus, it was concluded that TSPO is specifically a biomarker of pro-inflammatory processes [83], a finding which has also been validated more recently in a similar experimental approach [84].

These conclusions have also been supported by studies using TSPO ligands. In primary human microglia, pre-treatment with PK11195 inhibited LPS-activation, reduced COX2 and TNF-α production, as well as calcium influx compared with vehicle treated controls [85]. Importantly, the morphology of microglia, which also reflects functional state, was similar to controls, thereby demonstrating the modulatory effect of PK11195 on microglial function and morphology [85]. In rat primary microglia, PK11195 has also been shown to inhibit increases in NO after LPS stimulation [86]. In primary mouse microglia, Etifoxine and PK11195 pre-treatment before toll-like receptor ligand activation were able to reduce microglial activation and production of TNF-α, CCL2 and IL-6 [87]. Vinpocetine, a specific TSPO ligand, was also shown to inhibit the activation and proliferation of LPS-stimulated or oxygen-glucose deprived BV2 cells compared to untreated controls. Furthermore, Vinpocetine was found to reduce the release of NO, IL-1β, IL-6 and TNF-α, whilst simultaneously suppressing NF-κB and AP-1 upregulation, which are important signalling transduction pathways in the inflammatory response [88]. 

Very recently, these findings were replicated in microglial cells using new generation TSPO binding agents. BV2 cells stimulated with LPS in the presence of TSPO ligands 2-Cl-MGV-1, MGV-1 and PK11195 all were able to mitigate COX2, iNOS and NO [89]. In a separate study using BV2 cells, assessing M1/M2 modulation, 2-Cl-MGV and MGV-1 suppressed LPS-induced cytokines IL-6, IL-1β, TNF-α and IFN-γ, though had no effect on IL-10 or IL-13 levels. These ligands also decreased NF-κB p65 activity and decreased ROS production as measured through cardiolipin content [90]. 

Not all studies have demonstrated the same immunomodulatory effect of TSPO ligands in pro-inflammatory suppression. PK11195 and Ro5-4864 treatment in rat primary microglia was found to increase the rate of phagocytosis, cell proliferation, ROS generation (also acting through NADPH oxidase), and IL-1β secretion, thereby increasing the pro-inflammatory capacity of microglia [91]. In TSPO knockdown BV2 cells challenged with LPS, increased ROS production, TNF-α expression and microglial proliferation rate has also been reported [92]. This same effect was also reported using the RAW 264.7 macrophage cell line, where TSPO knockdown cells exposed to Hemin activation increased TNF-α and IL-6 release compared to scramble controls [93]. In human cells, differential effects have also been reported. In human monocyte-derived macrophages, induced to an M1 state, TSPO mRNA expression was reduced compared to controls. There was also no difference in TSPO expression in M2 stimulated macrophages compared to control [94]. In human primary monocyte-derived macrophages, stimulated with LPS/IFN-γ, decreased TSPO gene expression and radioligand binding has also been reported in another study [95]. These results may reflect the known complexity of the functional and activation states of microglial cells, that may also be species dependent and dependent on the concentration of ligand applied. 

### 3.2. Molecular Pathways of TSPO Immunomodulation

At the molecular level, the TSPO activation pathway interacts with downstream inflammatory effectors. Ro5-4864 application in THP-1 monocytes and bone marrow-derived macrophages has been shown to inhibit NLRP3 inflammasome complex activation and assembly after ATP stimulation, accompanied by reduced caspase-1 activation, IL-1β and IL-18 secretion [20]. TSPO transcription and expression is driven by c-Jun and STAT3 through the MAPK and PKCε signal transduction pathways, all of which are key to the induction of an inflammatory response [96,97,98]. ROS is also intrinsically linked to this pathway, activating PKCε-dependent pathways and c-Jun to control TSPO transcription [99]. PK11195 has also been shown to modulate genes and transcription factors in U118MG glioblastoma cells, particularly those related to cell viability, cell death, proliferation, and tumorigenesis, therefore showing that TSPO is involved in mitochondria-nuclear signalling and can modulate gene expression [22]. In the human macrophage cell line THP-1, exposure to Midazolam, a TSPO binding agent, suppressed LPS-induced inflammatory responses, including IL-6 expression, NO and NF-κB and MAPK activation. This was specific for TSPO, as TSPO knockout cells did not show this effect [100]. Hence, TSPO interacts with inflammation pathways at the transcriptional level, manifesting in the immunomodulatory effects described in this review.

### 3.3. In Vivo Immunomodulation of TSPO Ligands

Using in vivo models, TSPO ligands have been shown to modulate inflammation after injury or in certain disease states, thereby demonstrating neuroprotective properties (Table 2). In the earliest study examining this effect, Zavala et al. [101] demonstrated that administration of PK11195 and Ro5-4864 in mice impaired the release of IL-1β, TNF-α and IL-6 by peritoneal and spleen macrophages, accompanied by impaired oxidative respiratory burst of these macrophages. Since this finding, there have been several other studies using different disease and injury models demonstrating analogous effects. In an excitotoxic rat model of Huntington’s disease, PK11195 administration reduced the number of activated microglia after injury, and also reduced IL-1β, IL-6, TNF-α and iNOS mRNA expression after injury. This was also accompanied by a reduction in 4-HNE (lipid peroxidation) and 8-OHdG (oxidative DNA damage), alongside increased neuronal cell survival in the striatum near the site of injection, thereby conferring an anti-inflammatory and neuroprotective effect [102]. A similar phenomenon was also demonstrated with 3 novel pyrazolopyrimidine TSPO ligands; DPA-713, DPA-714 and propargyl-DPA in a quinolinic acid model of excitotoxic neurodegeneration. All pyrazolopyrimidine ligands decreased microglial activation and promoted neuronal survival in the injected striatum compared to vehicle controls [103]. 

To emphasise the neuroprotective potential of a variety of TSPO ligands, several other studies have demonstrated improved progression, symptoms and functional recovery in various disease and injury models following ligand administration [104,105]. Etifoxine was found to promote greater regeneration of myelinated axons compared to vehicle controls 2 weeks after sciatic nerve crush injury. This was accompanied by increased neuronal survival, reduced activation of macrophages, and reduced pro-inflammatory cytokine secretion. Ultimately, this manifested in the recovery of sensory and motor functions [106]. Etifoxine administration has also been found to improve scores in behavioural testing and rescues from neuronal degeneration in a rat model of traumatic brain injury, accompanied by reduced microglial activation and reduced cortical concentrations of IL-1α, IL-1β, IL-6, CCL2 and TNF-α [107]. In an Alzheimer’s disease mouse model, application of Ro5-4864 attenuated the accumulation of amyloid beta plaques in the hippocampus and decreased microglial activation. Importantly, this was accompanied by improved behaviour and cognition [108]. In a study using a retinal degeneration mouse model, application of XBD173, another new generation TSPO ligand prevented microglial reactivity in the retina when exposed to injury and reduced both IL-6 and CCL2 gene expression. These findings were accompanied by prevention of retinal degeneration after injury, hence drastically inhibiting the progression of disease [109]. XBD173 has also been shown to decrease pro-inflammatory cytokines and delay Multiple Sclerosis progression and symptoms in an experimental autoimmune encephalomyelitis (EAE) mouse model [110], and also demonstrated efficacy in attenuating microglial activation and neuronal loss in an MPTP Parkinson’s disease model [111].

## 4. Future Directions

This review has presented the growing body of literature investigating the role of TSPO in inflammatory responses and in mitochondrial processes, where function has largely been explored through pharmacological studies. Whilst the diazepam derivative Ro5-4864 is regarded as selective for TSPO, careful reading of earlier studies reports the possibility of some remnant binding to GABA-ergic sites [112,113]. Hence, a degree of caution is necessary in the interpretation of in vitro observations where high concentrations of Ro5-4864 have been used. Whilst for PK11195 no other obvious binding site is known, the compound might insert itself into the lipid bilayers [114,115], an interaction that speculatively may contribute to the recent observation that the residence time of PK11195, rather than merely its binding affinity to TSPO, may determine its physiological activity [116]. Nonetheless, the continual study of novel TSPO binding agents will be important for the development of therapeutic compounds for a range of disorders. The therapeutic potential of TSPO ligands have already been demonstrated in combination with photodynamic [117] and sonodynamic therapy [118], where the ability of TSPO to modulate ROS and apoptosis mechanisms can be exploited. Whilst much has been learned through pharmacological targeting of TSPO, deletion studies of TSPO are comparatively lacking. Further studies using TSPO deficient cells and animal models under stress conditions, for example, after irradiation, are needed to clarify the exact role of TSPO in oxidative stress responses and microglial responses. Further studies using various experimental approaches are also required to address the discrepancies in data looking at ROS and energy metabolism, where pharmacological evidence and genetic deletion studies give sometimes inconsistent results. This may be owing to the differences between TSPO knockdown versus knockout, and the range of ligand concentrations used in studies. Studies using TSPO knockout material are also necessary in order to determine the specific effects or off-target effects of novel compounds. 

The most widely studied context of TSPO has been centred around pro-inflammatory responses specifically. However, there are studies that demonstrated no difference or even decreases in TSPO expression after exposure to a pro-inflammatory stimulus in vitro [94,95]. It is also interesting that in a TSPO deficient mouse model, the ability to mount a local microglial response to nerve cell injury was not compromised [41], and in a more recent TSPO knockout model, TSPO deficiency did not affect microglial number or morphology in models of retinal degeneration [119]. The complex spectrum from pro- to anti-inflammatory processes in microglia has not yet been fully characterised, and the extent to which TSPO expression levels may provide an indication of the balance between pro- and anti-inflammatory tissue responses remains open. The terms “microglial activation” and “neuroinflammation” are perhaps too broad and require comprehensive assessment in response to specific stimuli, and may involve systems biological approaches going forward [120]. 

Even in vivo, PET studies have also reported a decrease in TSPO binding in certain disorders. For example, in a mouse schizophrenia model, TSPO radioligand binding was decreased in the prefrontal cortex, which did not match the concomitant increases in inflammatory cytokine levels [34]. In recent studies, evidence has confirmed that there is constitutive TSPO expression in cell types other than activated microglia in the brain. This includes, for example, neural stem cells, tanycytes, and most abundantly, vascular endothelial cells across the brain [26,121,122,123]. This has brought into question the approaches used for TSPO signal quantification in molecular imaging studies, and also raises questions as to the interpretation of imaging studies where a downregulation of TSPO is reported. Notably, when reductions below normal levels are observed, this may reflect a reduction in constitutively expressed TSPO which appears to be mainly located in the vasculature. Therefore, investigating other known cell types of TSPO expression over longer periods of time will be useful in order to ascertain whether the cumulative change in the regional expression levels of TSPO are due to a change in the number of cells expressing inducible TSPO, or whether indeed a downregulation of TSPO takes place in individual cells in which TSPO increases had been induced before. Furthermore, investigating other known cell types of TSPO expression will also be useful for future investigations into the functions of TSPO. This may be particularly pertinent given that the cell types of highest TSPO expression appear to be mitotically active, proliferative cells, further pointing to a fundamental role for TSPO in energy metabolism.

## Figures and Tables

**Table 1:** Summary of in vitro and in vivo experimental data describing the role of TSPO in mitochondrial processes.

Model	Treatment/Ligands (Concentration)	Outcome	Reference
In vitro			
Mouse GL261 glioma cells	TSPO KO	↑ mitochondrial fragmentation, fission proteins, ROS, glycolysis ↓ Complex I activity, MMP, global ATP production, basal and maximal mitochondrial respiratory capacity	Fu et al. (2019)
Human C20 microglia	TSPO KO	↓ MMP, basal and maximal respiration, ATP-related oxygen consumption	Milenkovic et al. (2019)
Mouse primary microglia	TSPO KO	↓ mitochondrial ATP production, basal oxygen consumption rate	Banati et al. (2014)
Mouse BV2 microglia	TSPO KD and scramble control cells LPS + Ro5-4864 (100 nM), PK11195 (100 nM), XBD173 (1 or 10 μM)	TSPO KD ↓ MMP, proliferation ↑ non-mitochondrial respiration, proton leak Control cells, Ro5-4864 and PK11195 ↑ basal respiration, ATP-related oxygen consumption ↓ spare respiratory capacity TSPO KD, control cells, Ro5-4864, PK11195 ↑ MMP TSPO KD, Ro5-4864 ↑ maximal respiration and proton leak TSPO KD, PK11195, XBD173 ↑ coupling efficiency	Bader et al. (2019)
Human U118MG cells	TSPO KD or Sodium nitroprusside + PK11195 (25 μM)	↑ metabolic activity ↓ cell death, MMP collapse	Shargorodsky et al. (2012)
Human U118MG cells	TSPO KD or CoCl_2_ + PK11195 (25 μM)	↓ apoptosis, MMP collapse, ROS	Zeno et al. (2009)
Mouse embryonic fibroblasts, canine mammary gland epithelia	TSPO KD or overexpression	TSPO KD ↑ ATP production, GSH, LC3B-II ↓ ROS TSPO overexpression ↑ ROS ↓ GSH	Gatliff et al. (2014)
HeLa cells	Bcl-2 KD + PK11195 (100 μM)	↓ mitophagy, F1Fo-ATPsynthase	Seneviratne et al. (2012)
Mouse peritoneal macrophages	PK11195, Ro5-4864 (1 μM)	PK11195 ↓ basal ROS Ro5-4864 ↑ basal ROS	Kupa et al. (2017)
Mouse BV2 microglia	PK11195, Ro5-4864, PPIX (10 nM)	↑ ROS	Jayakumar et al. (2002)
Rat C6 and Human T98G cells	PK11195, Ro5-4864 (10 nM)	↑ number of mitochondria, dividing mitochondria	Shirashi et al. (1991)
Mouse C1300 neuroblastoma cells	PK11195, Ro5-4864 (1 fm – 1 μM)	↓ oxygen consumption	Larcher et al. (1989)
Rat isolated liver, kidney, adrenal mitochondria	PK11195, Ro5-4864 (1 μM)	↓ mitochondrial respiratory control ratio	Hirsch et al. (1988)
Human T98G cells	Glucose deprivation + Ro5-4864 (10 nM-10 μm)	↑ cell viability, MMP preservation ↓ ROS	Baez et al. (2017)
Human SH-SY5Y neuroblastoma cells	Mutant Tau cells + imidazoquinazolinones compounds 2a and 2b (10 nM)	↑ ATP levels, MMP	Grimm et al. (2019)
Human SH-SY5Y neuroblastoma cells	APP overexpression + imidazoquinazolinones compounds 2a and 2b (10 nM)	↑ mitochondrial respiration ↓ ROS, oxidative injury, cell death, Aβ levels	Lejri et al. (2019)
Human Jurkat cells	TSPO transfection	↑ mitochondrial ATP production, cell proliferation	Liu et al. (2017)
Human Jurkat cells	TSPO transfection + UV exposure	↓ superoxide, caspase 3 activity, MMP ↑ apoptosis resistance	Stoebner et al. (2001)
In vivo			
Drosophila Aβ-42 induced neurodegeneration	TSPO KO	↓ radiation-induced apoptosis, mitochondrial respiration, OXPHOS enzyme activity ↑ H_2_O_2_ resistance	Lin et al. (2014)
Drosophila model of alcohol dependence	Conditional TSPO KO in neurons	↑ ROS (males only) ↓ caspase 3/7 activity	Lin et al. (2015)
Rat hypercholesterolemic rats	Myocardial ischemia-reperfusion + Ro5-4864 (10 mg/kg)	↑ calcium retention, respiratory control ratio	Musman et al. (2017)
Rat cortical trauma injury	Ro5-4864, (5 mg/kg, repeat)	↓ mitochondrial ultrastructural damage, metabolic damage ↑ neurological recovery	Soustiel et al. (2011)
Rat postischemia reperfusion, heart tissue	Ro5-4864 (16 μmol/L, 32 μmol/L, 64 μmol/L	↓ ROS, NADPH oxidase ↑ functional recovery, complex I and III activity	Xiao et al. (2010)
Rat cortical infarction	2-CI-MGV (7.5 mg/kg, repeat)	↑ MMP, cognitive impairments, neuronal survival ↓ cytochrome c, Iba1+ microglia	Chen et al. (2017)
Mouse spinal cord injury	ZBD-2 (10 mg/kg, repeat)	↓ serum MDA, iNOS ↑ SOD, neuronal survival	Cheng et al. (2016)

Abbreviations: KO=knockout, ROS=reactive oxygen species, MMP=mitochondrial membrane potential, KD=knockdown, Aβ=amyloid beta, APP=amyloid precursor protein, GSH=glutathione, MDA=malondialdehyde, iNOS=inducible nitric oxide synthase, SOD=superoxide dismutase, OXPHOS=oxidative phosphorylation.

**Table 2:** Summary of in vitro and in vivo experimental data describing the role of TSPO in inflammatory processes.

Model	Treatment/Ligands (Concentration)	Outcome	Reference
In vitro			
Human C20 microglia	TSPO KD or IL-1β/IL-1β+IFN-γ + PK11195, Ro5-4864, Etifoxine, XBD173 (all 100 nM)	IL-1β/IL-1β+IFN-γ ↑ TSPO mRNA Etifoxine, XBD173 ↓ IL-8 Ro5-4864, PK11195, Etifoxine, XBD173 ↑ IL-4 ↓ ROS TSPO KD ↑ IL-8 ↓ IL-4	Da Pozzo et al. (2019)
Mouse RAW 264.7 macrophages	TSPO KD or Hemin activation + Ro5-4864 (5 and 10 μM)	Ro5-4864 ↓ TNF-α, IL-6 TSPO KD ↑ IL-6, TNF-α	Bonsack et al. (2016)
Mouse BV2 microglia	LPS + TSPO KD	↑ ROS, TNF-α, proliferation rate	Wang et al. (2014)
Mouse BV2 microglia	LPS + TSPO KD/overexpression, PK11195, Ro5-4864 (0.1 or 10 μM)	LPS + TSPO overexpression/ligands ↑ M2 related genes ↓ NF-κB activity, IL-6, TNF-α LPS +TSPO KD ↑ IL-6, TNF-α, NO	Bae et al. (2014)
Mouse primary microglia and bone-marrow derived macrophages	LPS/IL-4 polarisation	LPS ↑ TSPO expression	Pannell et al. (2019)
Human monocyte-derived macrophages and microglia	LPS/IL-4/IL-13	LPS ↓ TSPO mRNA, TSPO radioligand binding	Owen et al. (2017)
Mouse primary microglia	LPS/IL-4 polarisation	LPS ↑ TSPO mRNA	Beckers et al. (2018)
Human monocyte-derived macrophages	LPS/IFN-γ stimulation	↓ TSPO mRNA	Narayan et al. (2017)
Mouse BV2 microglia	LPS + 2-MGV-1, MGV-1, PK11195 (25 μM)	↓ COX2, iNOS, NO	Azrad et al. (2019)
Mouse BV2 microglia	LPS + 2-MGV-1, MGV-1 (25 μM)	↓ IL-6, IL-1β, TNF-α, IFN-γ, ROS, NF-κB p65 activity	Monga et al. (2019)
Human THP-1 monocytes/macrophages, mouse primary bone marrow-derived macrophages	LPS + ATP + Ro5-4864 (50 μM)	↓ NLRP3 inflammasome activation/assembly, caspase-1, IL-1β, IL-18, ROS, MMP depolarization	Lee et al. (2016b)
Mouse BV2 microglia	LPS or oxygen-glucose deprivation + Vinpocetine (20 and 50 μM)	↓ microglial activation and proliferation, NO, IL-6, IL-1β, TNF-α, NF-κB, AP-1	Zhao et al. (2011)
Human THP-1 macrophages	LPS + Midazolam (15 μM)	↓ IL-6, NO, NF-κB, MAPK	Horiguchi et al. (2019)
Rat primary microglia	LPS + PK11195 (100 μM)	↓ NO	Wilms et al. (2003)
Human primary microglia	LPS + PK11195 (1 μM or 50 μM)	↓ COX2, TNF-α, calcium influx, microglial activation	Choi et al. (2002)
Rat primary microglia	PK11195, Ro5-4864 (1nm-100 nM)	↑ phagocytosis, cell proliferation, ROS, NADPH oxidase, IL-1β, microglial activation	Choi et al. (2011)
Mouse primary microglia	TLR ligand activation + PK11195, Etifoxine (50 μM)	↓ TNF-α, IL-6, CCL2	Lee et al. (2016)
In vivo			
Mouse 3xTg-AD Alzheimer’s Disease	Ro5-4864 (3 mg/kg, repeat)	↓ Aβ plaques, Iba1+ microglia ↑ behaviour and cognition	Barron et al. (2013)
Mouse primary peritoneal macrophages	LPS + Ro5-4864 (1 mg/kg)	↓ IL-1β, TNF-α, IL-6, oxidative metabolism	Zavala et al. (1990)
Rat excitotoxic Huntington’s Disease	Quinolinic acid + PK11195 (5 nM)	↓ Iba1+ microglia, IL-1β, IL-6, TNF-α, iNOS, 4-HNE, 8-OHdG ↑ neuronal survival	Ryu et al. (2005)
Rat excitotoxic neurodegeneration	Quinolinic acid + DPA-713, DPA-714 and propargyl-DPA (5 nM)	↓ OX-42+ microglia ↑ neuronal survival	Leaver et al. (2012)
Mouse MPTP Parkinson’s Disease	XBD173 (50 mg/kg, repeat)	↑ neuronal survival, dopamine, motor function, IL-10 ↓ Iba1+ microglia, COX2, CXCL10	Gong et al. (2019)
Mouse EAE model	XBD173 (10, 20, 30 mg/kg, repeat)	↓ IL-6, TNF-α and IL-17, clinical EAE score ↑ MBP expression, motor function	Leva et al. (2017)
Mouse retinal degeneration	XBD173 (10 mg/kg, repeat)	↓ Iba1+ microglia, IL-6, CCL2, retinal degeneration	Scholz et al. (2015)
Rat traumatic brain injury	Etifoxine (50 mg/kg, repeat)	↑ behaviour and sensorimotor function ↓ CD68+ microglia, neuronal degeneration, IL-1α, IL-1β, IL-6, TNF-α, CCL2	Simon O’Brien et al. (2016)
Rat sciatic nerve crush injury	Etifoxine (50 mg/kg, repeat)	↑ myelination, neuronal survival, sensory and motor function ↓ macrophage activation (OX-42+), IL-6, TNF-α, IL-1β	Girard et al. (2008)

Abbreviations: KD=knockdown, NO=nitric oxide, LPS=lipopolysaccharide, MMP=mitochondrial membrane potential, ROS=reactive oxygen species, EAE=experimental autoimmune encephalomyelitis, MBP=myelin basic protein.
